# *Mycoplasma hyopneumoniae* surface-associated proteases cleave bradykinin, substance P, neurokinin A and neuropeptide Y

**DOI:** 10.1038/s41598-019-51116-w

**Published:** 2019-10-10

**Authors:** Veronica Maria Jarocki, Benjamin Bernard Armando Raymond, Jessica Leigh Tacchi, Matthew Paul Padula, Steven Philip Djordjevic

**Affiliations:** 1 ithree institute, University of Technology Sydney, PO Box 123, Broadway, NSW 2007 Australia; 2 Proteomics Core Facility, University of Technology Sydney, PO Box 123, Broadway, 2007 NSW Australia

**Keywords:** Proteases, Bacterial pathogenesis, Proteomics

## Abstract

*Mycoplasma hyopneumoniae* is an economically-devastating and geographically-widespread pathogen that colonises ciliated epithelium, and destroys mucociliary function. *M. hyopneumoniae* devotes ~5% of its reduced genome to encode members of the P97 and P102 adhesin families that are critical for colonising epithelial cilia, but mechanisms to impair mucociliary clearance and manipulate host immune response to induce a chronic infectious state have remained elusive. Here we identified two surface exposed *M. hyopneumoniae* proteases, a putative Xaa-Pro aminopeptidase (MHJ_0659; PepP) and a putative oligoendopeptidase F (MHJ_0522; PepF), using immunofluorescence microscopy and two orthogonal proteomic methodologies. MHJ_0659 and MHJ_0522 were purified as polyhistidine fusion proteins and shown, using a novel MALDI-TOF MS assay, to degrade four pro-inflammatory peptides that regulate lung homeostasis; bradykinin (BK), substance P (SP), neurokinin A (NKA) and neuropeptide Y (NPY). These findings provide insight into the mechanisms used by *M. hyopneumoniae* to influence ciliary beat frequency, impair mucociliary clearance, and initiate a chronic infectious disease state in swine, features that are a hallmark of disease caused by this pathogen.

## Introduction

*Mycoplasma hyopneumoniae* is the etiological agent of porcine enzootic pneumonia, a highly infectious and globally distributed swine respiratory disease. Symptoms of enzootic pneumonia include growth rate retardation, reduced feed conversion, and higher susceptibility to secondary bacterial^1^ and viral infections, including porcine reproductive and respiratory syndrome virus (PRRSV), swine influenza virus (SIV) and porcine circovirus type 2 (PCV2)^2,3^. Many broad-spectrum antibiotics are used to treat *M. hyopneumoniae* infections specifically, but a greater number of antibiotics are used to prevent polymicrobial respiratory infections^4^. Thus *M. hyopneumoniae* is a significant contributor to antibiotic use in swine production. In 1991, *M. hyopneumoniae* caused an estimated $1 billion economic loss in the USA alone^5^. This estimate did not account for the impact incurred by animal waste containing large quantities of multiple antibiotic resistant bacterial populations^6^. Animal waste including pig effluent is used as organic fertiliser on agricultural lands, particularly in China^7^, the world’s largest producer of pork. While no current economic burden estimates have been published, a 2006 survey of nineteen large US pig production companies ranked the estimated losses associated with *M. hyopneumoniae* alone and *M. hyopneumoniae* in conjunction with PRRSV among the top challenges with the highest estimated average loss for all pigs^8^.

Bacterin vaccines are used to control *M. hyopneumoniae* in conjunction with antibiotics; however, their efficacy is limited due to a minimal reduction in pathogen transmission and high production cost^9^. There is a need to enhance our understanding of *M. hyopneumoniae* pathogenesis to develop more efficacious vaccines and therapeutics that seek to eradicate this pathogen by preventing colonisation of the respiratory tract and reducing reliance on antibiotics.

The mucociliary escalator is a major innate barrier to all infectious respiratory microorganisms. It lines the respiratory tract and is composed of mucus-secreting goblet cells and ciliated epithelium. Mucus traps inhaled particles that are then propelled to the pharynx by the synchronised beating of cilia to be either swallowed or expectorated^10^. *M. hyopneumoniae* avoids mucociliary clearance by disrupting the mucociliary escalator by initiating ciliostasis, loss of cilia function, and epithelial cell death. However, these sequelae are poorly understood^11^. Cilioinhibitory factors deployed by other respiratory pathogens to disrupt the mucociliary system, such as the toxin pneumolysin of *Streptococcus pneumoniae*^12^ or the low molecular weight glycopeptides produced by *Haemophilus influenzae*^13^, have not been described for *M. hyopneumoniae*. The human respiratory pathogen, *Mycoplasma pneumoniae*, causes allergic-type inflammation by secreting the community-acquired respiratory distress syndrome (CARDS) toxin^14^, though homologues of this toxin have not been found in *M. hyopneumoniae*. A recent study demonstrated that *M. hyopneumoniae* signal peptidase I is cytotoxic to mammalian cells^15^, however this protease is not surface expressed or secreted^16,17^. While mycoplasmas can cause some direct tissue damage through the production of the metabolic by-product hydrogen peroxide^18^, this is not necessarily linked with pathology. For example, mutants of *Mycoplasma gallisepticum*, a major respiratory pathogen of poultry that are incapable of producing hydrogen peroxide, are virulent in a native host pathogenesis model^19^.

Many bacterial species are known to manipulate host defences by actively attracting immune effector cells to the site of infection, because host-derived proteases, released by neutrophils and macrophages, are often more efficient at degrading extracellular matrix (ECM) components than bacterial-derived proteases^20^. Host-induced ECM proteolysis represents a mechanism to source nutrients, a process that is vital for genome-reduced organisms, such as *M. hyopneumoniae*, unable to synthesise amino acids, nucleotides, fatty acids and other macromolecular building blocks^21,22^.

Mycoplasmas adhere to respiratory epithelium and elicit many defence mechanisms. These include the induction of pro-inflammatory cytokines such as tumour necrosis factor alpha (TNFα), interleukin (IL) 1β, IL6, and IL8, stimulation of lymphocytes, and increase the cytotoxicity of macrophages and natural killer (NK) cells^23^. Non-self-recognition also up-regulates several other respiratory defence mechanisms. Antimicrobial peptides (AMPs), such as lactoferrin, lysozyme, and cathelicidins are secreted from respiratory epithelium and not only directly destroy pathogens but also act as effector molecules regulating both innate and adaptive immune systems^24^. Inflammation is intensified by microbe-induced activation of enzymatic cascades, including the kallikrein/kinin system which releases bradykinin (BK), a potent bronchoconstrictor and pro-inflammatory peptide^25^. Neurogenic inflammation also plays a significant role in the innate immune response to infections, with non-myelinated C-fibres innervating the majority of the lung^26^. These fibres secrete neuropeptides such as neuropeptide Y (NPY), substance P (SP), and neurokinin A (NKA), the two latter of which are similar to AMPS in both structure and function^27^, and are known to stimulate mucus secretion and increased ciliary beat frequency (CBF)^28,29^.

Despite fostering a formidable immune response, *M. hyopneumoniae* is associated with chronic illness. Maintaining a balance of contrasting immunologic responses is therefore likely to impact virulence and disease progression. Host effector molecules and their receptors are susceptible to proteolytic modifications by bacterial proteases that render them either active or inactive^20^. Despite evolving via a process of genome decay, *M. hyopneumoniae* has retained the genetic capacity to express several putative proteases, five of which were observed to be overrepresented in pathogenic *M. hyopneumoniae* strains^17^, yet how these may affect their host has not been fully explored. For example, proteolytic activity against kallikrein-kinin system substrates, such as BK, has been demonstrated in *M. hyopneumoniae* and other mycoplasmas, however the proteases behind this activity have only been speculated on^30,31^. Here we show using several complementary, orthogonal methods that two proteases - MHJ_0659, a putative Xaa-Pro aminopeptidase (PepP), and MHJ_0522, a putative oligoendopeptidase F (PepF) - are exposed on the surface of *M. hyopneumoniae*. MHJ_0659 and MHJ_0522 were purified as recombinant polyhistidine fusion proteins using nickel-affinity chromatography and their enzymatic properties against substrates involved in mucociliary clearance, namely BK, SP, NKA, and NPY were assessed with different metal ion cofactors at different pHs using a MALDI-TOF/TOF MS/MS protocol.

## Results

### Identification and localisation of MHJ_0659 and MHJ_0522 in *M. hyopneumoniae*

**Figure 1:**
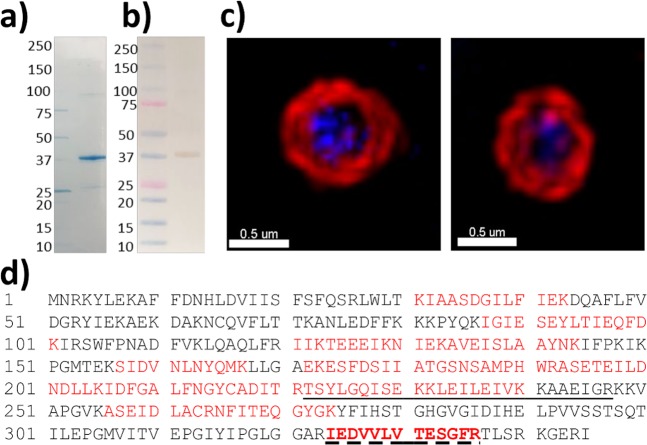
MHJ_0659 is detected on the surface of *M. hyopneumoniae*. (**a**) rMHJ_0659 resolves as a single band at approximately 39 kDa under denaturing conditions during SDS-PAGE. (**b**) Western blot demonstrating anti-MHJ_0659 sera specificity. (**c**) Two representative examples of *M. hyopneumoniae* cells stained with the nucleic acid stain DAPI (blue) and probed with both rabbit anti-MHJ_0659 serum and anti-rabbit antibodies conjugated to CFTM 568 (red). Bars represents 0.5 µm. (**d**) MHJ_0659 amino acid sequence. Tryptic peptides (red) identified by LC-MS/MS after the 39 kDa rMHJ_0659 protein was digested in-gel with trypsin. The underlined peptide was identified by LC-MS/MS after biotinylated surface proteins were recovered by avidin chromatography and digested with trypsin. Mascot identity score – 77.5; Mascot ion score – 89.4; Delta score 39.0; Peptide prophet – 97%. The dashed underlined peptide in bold was identified by LC-MS/MS after mild trypsin digestion of cell surface proteins. Mascot identity score – 68; Mascot ion score – 96.1; Delta score – 67.5; Peptide prophet – 99%.

**Figure 2:**
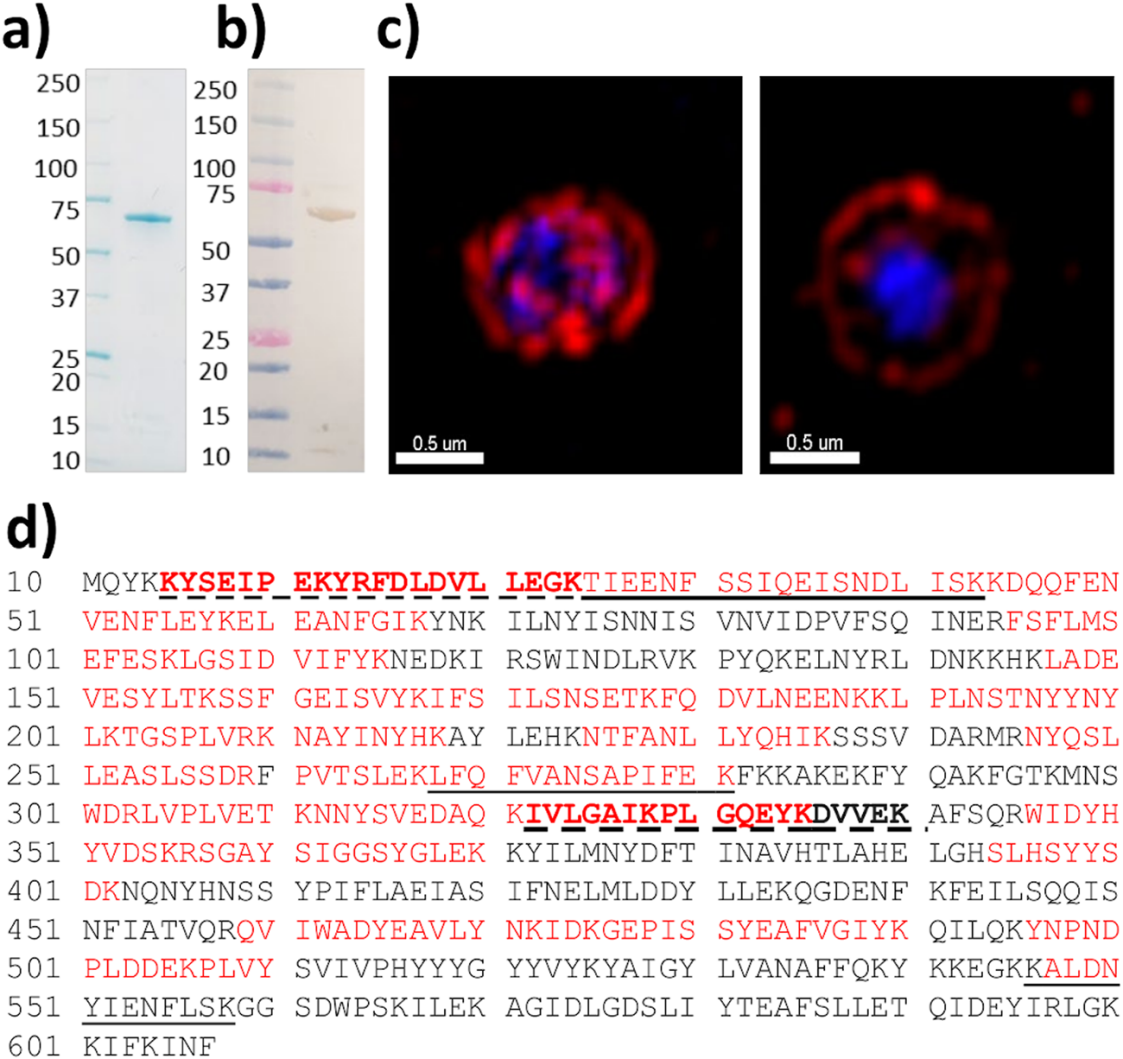
MHJ_0522 is detected on the surface of *M. hyopneumoniae* cells. (**a**) An SDS-PAGE gel of purified rMHJ_0522. The protein resolves as a monomer of approximately 70 kDa. (**b**) Western blot demonstrating anti-MHJ_0522 sera specificity. (**c**) Image illustrating the surface localisation of MHJ_0522 after whole *M. hyopneumoniae* cells were probed with rabbit anti-MHJ_0522 serum and anti-rabbit antibodies conjugated to CF^TM^ 568 (red). *M. hyopneumoniae* cells were also stained with the nucleic acid stain DAPI (blue). Bars represents 0.5 µm. (**d**) Tryptic peptides (red) that map to rMHJ_0522 provide 51% sequence coverage and confirm the identity of the purified recombinant protein. Tryptic peptides that are dash underlined and bold were those identified after mild trypsin digestion of the cell surface of *M. hyopneumoniae*. Tryptic peptides that are underlined were identified after biotinylated surface proteins were recovered by avidin chromatography and digested with trypsin.

Despite a PSORT prediction for a cytoplasmic localisation for MHJ_0659 and MHJ_0522, mild enzymatic cell shaving experiments using trypsin identified tryptic peptides matching MHJ_0659 (Fig. 1d; one peptide) and MHJ_0522 (Fig. 2d; three peptides). In a second, independent set of experiments in which freshly cultured *M. hyopneumoniae* cells were labelled with biotin, and surface-exposed biotinylated proteins were recovered using avidin chromatography, LC-MS/MS identified peptides that also mapped to MHJ_0659 (Fig. 1d; one peptide) and MHJ_0522 (Fig. 2d; two peptides). Specificity of anti-sera generated against MHJ_0659 and MHJ_0522 was checked via Western Blotting (Figs 1b and 2b). To confirm the surface localisation of both proteases, we labelled the surface of freshly cultured *M. hyopneumoniae* with rabbit anti-MHJ_0659 and anti-MHJ_0522 antibodies and detected them with anti-rabbit antibodies conjugated with CF^TM^ 568 (Figs 1c and 2c).

### Biochemical characterisation of functionally active rMHJ_0659

**Figure 3:**
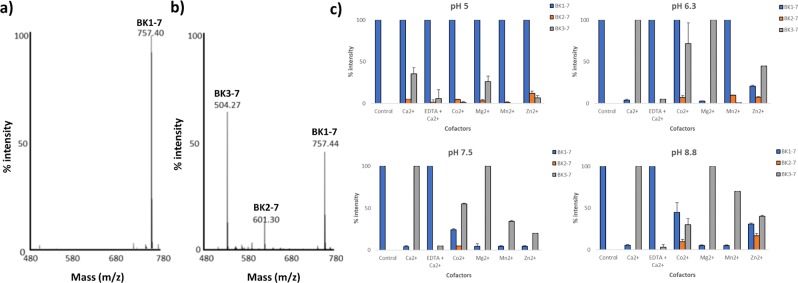
Cleavage of BK1-7 by rMHJ_0659 is influenced by pH and metal ion cofactors. (**a**) MALDI-TOF/MS spectra demonstrating a single peak at 757.4 Da that represents the mass of bradykinin fragment 1-7 (BK1-7). (**b**) MALDI-TOF/MS spectra of bradykinin peptide fragments and amino acids released after incubating BK1-7 with rMHJ_0659 for 1 hr in the presence of Co^2+^ at pH 7.5. Additional peaks observed at 601.3 Da (BK2-7) and 504.3 Da (BK3-7) represent the loss of an R residue, and loss of R and P residues respectively from the N-terminus of BK1-7. (**c**) At pH 5, the most prominent peak was the parent BK1-7. Smaller amounts of cleavage products BK2-7 and BK3-7 were detected at pH 5. At all pH levels tested, Ca^2+^ followed by Mg^2+^ produced the most intense peaks at a mass of 504.3 Da representing BK3-7. EDTA suppressed Ca^2+^ mediated activity at all pH levels tested. Control refers to BK1-7 in the absence of rMHJ_0659.

**Figure 4:**
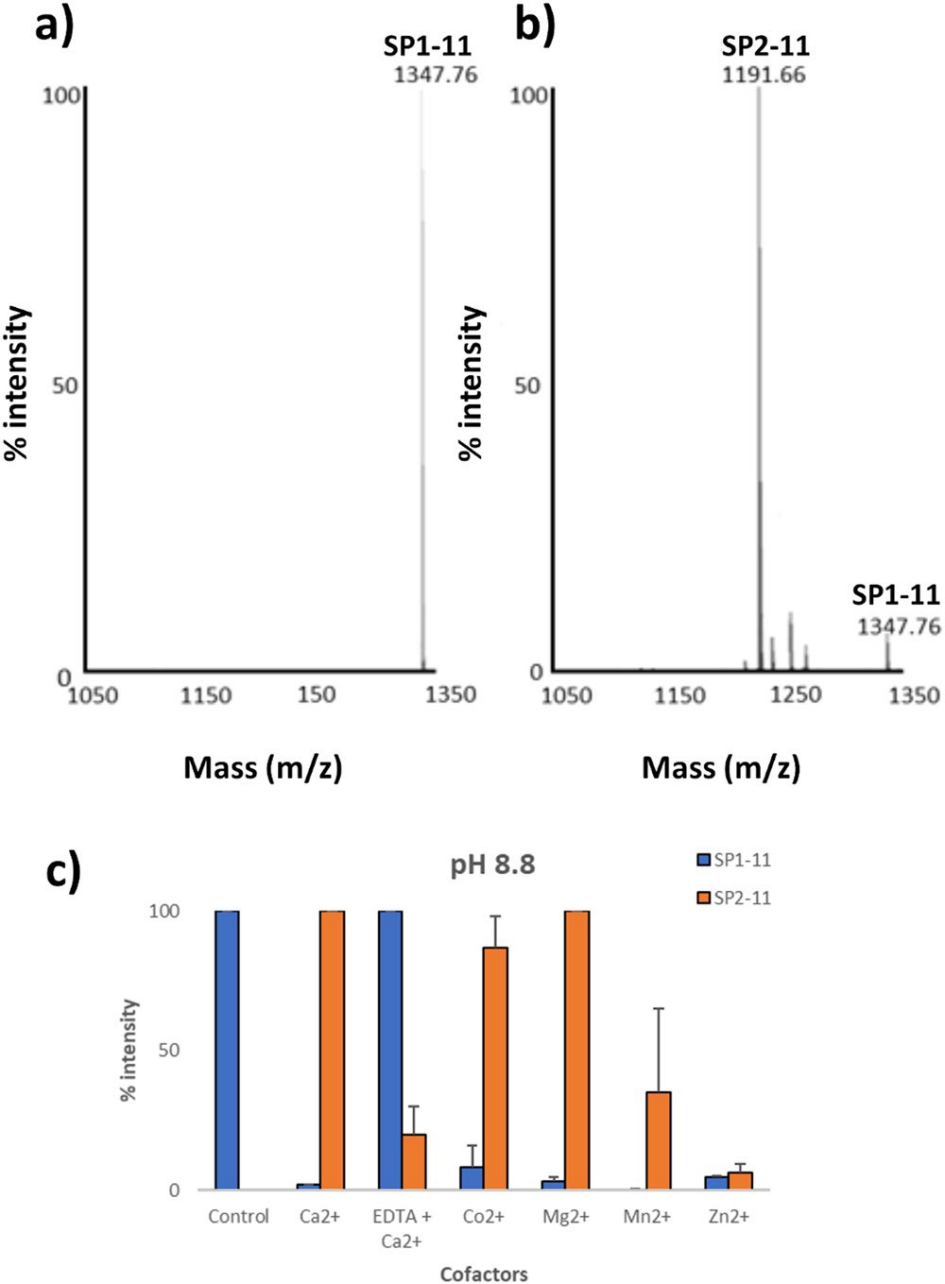
Cleavage of SP by rMHJ_0659 is influenced by pH and metal ion cofactors. (**a**) SP in the absence of rMHJ_0659 is represented as a single prominent peak at 1347.8 Da using MALDI-MS. (**b**) After SP was incubated with rMHJ_0659, the prominent peak was at 1191.7 Da. This ion represents the mass of SP minus the N-terminal arginine residue (SP fragment SP2-11). (**c**) The generation of SP2-11 occurred at pH 8.8 and was greatest in the presence of Ca^2+^ and Mg^2+^ and EDTA suppressed activity of Ca^2+^. Control refers to SP1-11 in the absence of rMHJ_0659.

**Figure 5:**
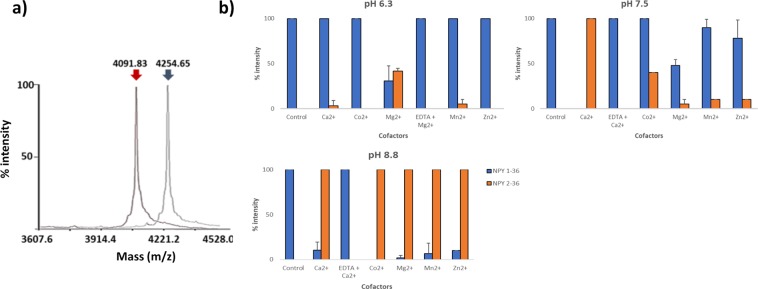
Cleavage of NPY by rMHJ_0659 is influenced by pH and metal ion cofactors. (**a**) Superimposed image of two MALDI-TOFMS spectras showing NPY in the absence of rMHJ_0659 represented by a peak at 4254.7 Da (blue arrow) and after incubation with rMHJ_0659 in the presence of Co^2+^ at pH 8.8 (red arrow). The peak at 4091.8 Da represents the mass of NPY after removal of tyrosine. (**b**) rMHJ_0659 cleaved NPY with the greatest efficiency at pH 8.8 in the presence of the metal ion cofactors Ca^2+^, Co^2+^, Mg^2+^, Mn^2+^, and Zn^2+^. EDTA suppressed Mg^2+^ (pH 6.3) and Ca^2+^ (pH 7.5 and pH 8.8) – mediated activity. Control refers to NPY1-36 in the absence of rMHJ_0659.

rMHJ_0659 was purified from *Escherichia coli* as a polyhistidine fusion protein and resolved as a single band with a mass of approximately 40 kDa by SDS-PAGE (Fig. 1a). This protein was recovered from the gel, digested with trypsin and confirmed to be MHJ_0659 by LC-MS/MS (46% sequence coverage) (Fig. 1d). rMHJ_0659 was able to remove the N-terminal penultimate proline from BK (Fig. 3), SP (Fig. 4) and NPY (Fig. 5) in a manner that is typical of a PepP protease.

### rMHJ_0659 cleaves bradykinin (BK)

The BK1-7 fragment [RPPGFSP] of bradykinin has a molecular mass of 757 Da. In the absence of rMHJ_0659, a single prominent peak at 757.40 Da representing the mass of BK1-7 was observed by MALDI-TOF/MS (Fig. 3a). Once BK1-7 was incubated with rMHJ_0659 two additional peaks were identified with masses of 601.30 Da and 504.27 Da (Fig. 3b). The 601.30 Da peak represents the mass of BK2-7 [PPGFSP] which arises by the removal of arginine from the N-terminus (R = 156.1 Da). The 504.27 Da peak equates to BK3-7 [PGFSP] and is the product of removal of the proline residue in position P2’ (P = 97.1 Da). The effect of divalent cofactors Ca^2+^, Co^2+^, Mg^2+^, Mn^2+^ and Zn^2+^ at pH of 5, 6.3, 7.3 and 8.8 on rMHJ_0659 activity was also assessed. rMHJ_0659 had the highest activity in the presence of Ca^2+^ at all pH levels tested. The metal chelating agent EDTA in the presence of Ca^2+^ suppressed activity at all pH levels tested (Fig. 3c).

### rMHJ_0659 cleaves substance P (SP)

SP is an undecapeptide [RPKPQQFFGLM-_NH2_] with a mass of 1347.63 Da. When rMHJ_0659 was incubated with SP for an hour at 37 °C, the N-terminal arginine residue was removed generating the fragment SP2-11 [PKPQQFFGLM-_NH2_] with a mass of 1191.66 Da (Fig. 4b). This cleavage only occurred at pH 8.8, with cofactor Ca^2+^ and Mg^2+^ producing the most intense peaks. EDTA in the presence of Ca^2+^ suppressed activity compared to Ca^2+^ alone (Fig. 4c).

### rMHJ_0659 cleaves neuropeptide Y (NPY)

NPY is a 36 amino acid peptide [^1^YPSK…RQRY^36^] with a molecular mass of 4254.70 Da. A single peak at 4091.83 Da representing NPY minus the N-terminal tyrosine residue (Y = 163 Da) was detected when NPY was incubated with rMHJ_0659 in the presence of various divalent metal cofactors across a pH range of 5 to 8.8 for 1 hour at 37 °C (Fig. 5a). The peak at 4091.83 Da was not evident when rMHJ_0659 was incubated with NPY at pH 5.0. Minor cleavage of NPY was evident at pH 6.3 in the presence of Mg^2+^, Ca^2+^ and Mn^2+^. At pH 7.5, the metal ion cofactor Ca^2+^ provided the most favourable conditions for cleaving NPY. rMHJ_0659 cleaved NPY with maximum efficiency at pH 8.8 in the presence of different metal ion cofactors. EDTA suppressed Mg^2+^ mediated activity at pH 6.3, and Ca^2+^ activity at pH 7.5 and 8.8 (Fig. 5b).

## Biochemical Characterisation of rMHJ_0522

**Figure 6:**
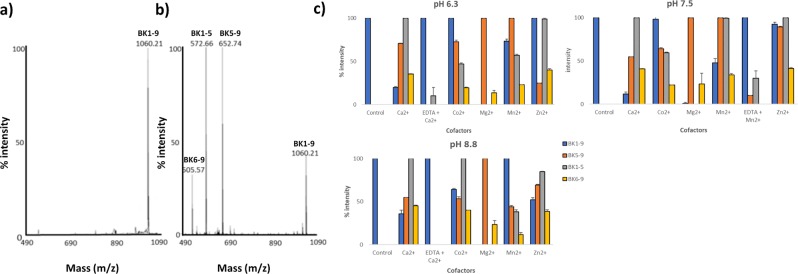
Cleavage of BK by rMHJ_0522 is influenced by pH and divalent metal cofactors. (**a**) Control spectra of BK in the absence of rMHJ_0522. (**b**) and (**c**) rMHJ_0522 produces fragments BK1-5, BK5-9, and BK6-9 at pH 6, 7.3 and 8.8 and in the presence of all cofactors tested. However, the intensity of each peak varies implying that at different pH levels and in the presence of various cofactors certain cleavages are more prevalent. EDTA suppressed Ca^2+^ mediated activity at pH 6.3 and pH 8.8, and suppressed Mn^2+^ mediated activity at pH 7.5. Control refers to BK1-9 in the absence of rMHJ_0522.

**Figure 7:**
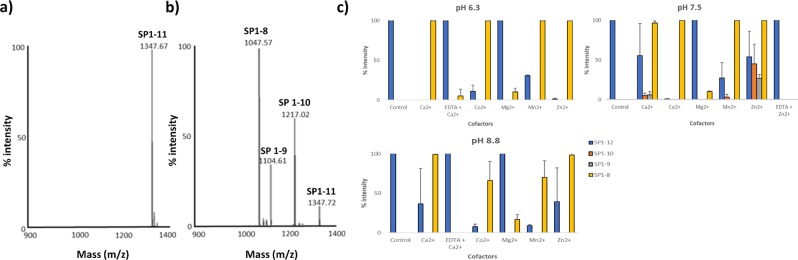
Cleavage of SP by rMHJ_0522 is influenced by pH and divalent metal cofactors. (**a**) Control spectra of SP illustrating a single peak at 1347.7 Da produced in the absence of rMHJ_0522. (**b**) Spectra of SP cleavage after incubation with rMHJ_0522 at pH 7.3 in the presence of Zn^2+^ illustrating generation of SP1-10 (RPKPQQFFGL = 1217.0 Da), SP1-9 (RPKPQQFFG = 1104.6 Da) and SP1-8 (RPKPQQFF = 1217.0 Da) (**c**) At pH 6, only the cleavage fragment SP1-8 was produced in the presence of each of the cofactors. At pH 7.3, the most common cleavage fragment was SP1-8. However, the presence of Zn^2+^ produced additional cleavage fragments SP1-9 and SP1-10, as did Ca^2+^, albeit at lower intensities. At pH 8.8 only SP1-8 was produced. However, the intensities for Co^2+^, Mg^2+^ and Mn^2+^ were lower. EDTA suppressed cofactor mediated activity across all pH levels tested. Control refers to SP1-11 in the absence of rMHJ_0522.

**Figure 8:**
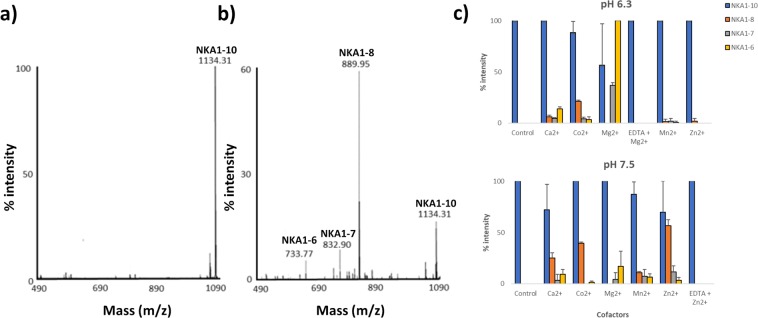
Cleavage of NKA by rMHJ_0522 is influenced by pH and divalent metal cofactors. At pH 6.3 rMHJ_0522 produced non-negligible fragments in the presence of Mg^2+^, Co^2+^ and Ca^2+^. At pH 7.3 and most intensely in the presence of Zn^2+^, rMHJ_0522 generated three cleavage fragments (NKA1-6, NKA1-7, and NKA1-8), with NKA1-8 being the most prominent. EDTA suppressed Mg^2+^ mediated activity at pH 6.3 and Zn^2+^ mediated activity at pH 7.5. Control refers to NKA1-10 in the absence of rMHJ_0522.

rMHJ_0522 (predicted MW of 71 kDa) resolves during SDS-PAGE as a single band with a mass of approximately 70 kDa (Fig. 2a). The protein band was digested with trypsin and confirmed to be rMHJ_0522 by LC-MS/MS (51% sequence coverage) (Fig. 2d). rMHJ_0522 cleaved full-length BK (Fig. 6), SP (Fig. 7) and NKA (Fig. 8). Unlike rMHJ_0659, rMHJ_0522 was not active at pH 5. rMHJ_0522 did not cleave BK1-7 nor did it cleave NPY, which is 36 amino acids in length. These data are consistent with rMHJ_0522 functioning as an oligoendopeptidase.

### rMHJ_0522 cleaves bradykinin (BK)

Full-length BK [RPPGFSPFR] has a molecular mass of 1060.21 Da. In the absence of rMHJ_0522, a single prominent peak at 1060.21 Da was observed in MALDI-TOF MS spectra (Fig. 6a). When BK was incubated with rMHJ_0522, three additional peaks were observed, 652.74 Da, 572.66 Da, and 505.57 Da (Fig. 6b). The peaks at 572.66 Da and 505.57 Da are products of a single cleavage event; 572.66 Da represents the mass of BK1-5 [RPPGF], and 505.57 Da represents the mass of BK6-9 [SPFR], indicating the cleavage event occurs at RPPGF↓SPFR. The peak at 652.74 Da equates to the mass of BK5-9 [FSPFR] thus represents another cleavage event at RPPG↓FSPFR. The effect of divalent cofactors Ca^2+^, Co^2+^, Mg^2+^, Mn^2+^ and Zn^2+^ and pH on the activity of rMHJ_0522 was determined. At pH 6.3, Mn^2+^ and Mg^2+^ produced the most intense peak for BK5-9, and both Ca^2+^ and Zn^2+^ produced the most intense peaks for BK1-5. A similar profile was seen at pH 7.3; only Mn^2+^ joined Ca^2+^ and Zn^2+^ in producing the most intense peak for BK1-5. In basic conditions (pH ≥ 7.5), only Mg^2+^ produced intense peaks for BK5-9, and Ca^2+^ and Co^2+^ produced the most intense peaks for BK1-5 (Fig. 6c). EDTA supressed Ca^2+^ mediated activity at pH 6.3 and pH 8.8, and Mn^2+^ mediated activity at pH 7.5 (Fig. 6c).

### rMHJ_0522 cleaves substance P (SP)

Full-length SP [RPKPQQFFGLM-_NH2_] has a molecular mass of 1347.67 Da and control experiments demonstrated a single ion peak at this mass (Fig. 7a). When SP was incubated with rMHJ_0522 additional peaks were observed at 1047.57 Da, 1104.61 Da and 1217.02 Da corresponding to the mass of SP fragments SP1-8 [RPKPQQFF], SP1-9 [RPKPQQFFG], and SP1-10 [RPKPQQFFGL], respectively (Fig. 7b). The most prevalent cleavage fragment generated at all pHs (6.3, 7.3 & 8.8) and cofactors (Ca^2+^, Co^2+^, Mg^2+^, Mn^2+^, and Zn^2+^) tested was SP1-8 (Fig. 7c), however at pH 7.3 in the presence of Zn^2+^ and Ca^2+^, SP1-9 and SP1-10 were also produced, albeit at lower intensities (Fig. 7c). This data indicates that rMHJ_0522 can produce cleavage events at RPKPQQFF**↓**G↓L↓M (first arrow indicates most prominent cleavage). EDTA suppressed Ca^2+^ mediated activity at pH 6.3 and pH 8.8, and Zn^2+^ mediated activity at pH 7.5 (Fig. 7c).

### rMHJ_0522 cleaves neurokinin A (NKA)

Full-length NKA [HKTDSFVGLM-_NH2_] has a molecular mass of 1134.31 Da and a single ion at that mass was detected in control experiments (Fig. 8a). When NKA was incubated with rMHJ_0522, additional peaks were observed at 733.77 Da, 832.90 Da and 889.95 Da corresponding to the mass of NKA fragments NKA1-6 [HKTDSF], NKA1-7 [HKTDSFV], and NKA1-8 [HKTDSFVG], respectively (Fig. 8b). At pH 6.3 and only in the presence of Ca^2+^, Co^2+^ and Mg^2+^ did rMHJ_0522 produce non-negligible cleavage fragments of NKA. NKA1-6 and NKA1-7 were the most common cleavage fragment in the presence of Mg^2+^ and NKA1-8 the most prominent in the presence of Co^2+^. At pH 7.3, Zn^2+^ was the most effective cofactor, producing NKA1-8 as the most predominant fragment. Unlike BK and SP, no activity against NKA was observed at pH 8.8. EDTA suppressed Mg^2+^ mediated activity at pH 6.3 and Zn^2+^ mediated activity at pH 7.5 (Fig. 8c).

### rMHJ_0659 and rMHJ_0522 share substrates and have the potential to cleave many biologically active proteins

rMHJ_0659 was shown to cleave at N-terminal penultimate prolines when the N-terminal amino acid was arginine (BK and SP), proline (BK second cleavage) and tyrosine (NPY). rMHJ_0522 cleaved BK at glycine and phenylalanine, SP at phenylalanine, glycine and leucine, and NKA at phenylalanine, valine, and glycine (Table 1). BK and SP were common substrates for both proteases. Interestingly, penultimate prolines are present at the N-terminal of many porcine innate immune system polypeptides, and the FXGLM-_NH2_ motif is common to all tachykinins, the largest family of neuropeptides (Table 2). We propose that these innate effector peptides and neuropeptides are potential substrates for PepP and PepF proteases on the surface of *M. hyopneumoniae*.

**Table 1:** Peptide substrates for rMHJ_0659 and rMHJ_0522.

Substrate	Cleavage site
Bradykinin	R**↓**P**↓**P-G↓F↓S-P-F-R
Substance P	R**↓**P-K-P-Q-Q-F-F↓G↓L↓M-_NH2_
Neurokinin A	H-K-T-D-S-F↓V↓G↓LM-_NH2_
Neuropeptide Y	Y**↓**P-S-K-P-D-N-P-G…

Bold arrows and underline arrows represent rMHJ_0659 and rMHJ_0522 cleavage sites respectively.

**Table 2:** Porcine innate immune system polypeptides that exhibit penultimate proline (left) residues and tachykinin C-terminal sequences (right).

Active Lung Proteins and Peptides	N-terminal Sequence	Tachykinins	C-terminal Sequence
Alveolar macrophage chemotactic factor	S**P**IEAA…	Endokinin A	…QFFGLM-_NH2_
Bradykinin	R**P**PGFS…	Hemokinin	…QFYGLM-_NH2_
Coagulation factor XII	I**P**PWKD…	Neurokinin A	…SFVGLM-_NH2_
Cathepsin B	L**P**KSFD…	Neurokinin B	…FFVGLM-_NH2_
Cathepsin K	T**P**DSID…	Neuropeptide γ	…SFVGLM-_NH2_
Growth hormone	F**P**AMPL…	Neuropeptide κ	…SFVGKM-_NH2_
Interleukin 2	A**P**TSSS…	Substance P	…QFFGLM-_NH2_
Interleukin 6	F**P**TPGR…		
Interleukin 13	G**P**VPPH…		
Lactoferrin	A**P**KKGV…		
Neuropeptide Y	Y**P**SKPD…		
Pulmonary surfactant-associated protein B	F**P**IPLP…		
Substance P	R**P**KPQQ…		

## Discussion

*M. hyopneumoniae* enters the porcine respiratory tract on mucosal droplets expelled during bouts of coughing from infected animals. In naïve animals, these droplets are inhaled and are likely to be captured by the mucociliary escalator that forms an effective barrier to invading pathogens. The ability of this porcine-specific pathogen to survive as a species is contingent on being able to colonise cilia that beat with a frequency of ~13 hertz in normal uninfected tissue and overcome the mucociliary escalator^32^. A toxin that can disrupt mucociliary clearance by slowing or blocking ciliary beat frequency has not been identified in *M. hyopneumoniae*, yet it is highly adept at colonising ciliary epithelium. *M. hyopneumoniae* devotes more than 5% of its reduced genome^23^ to encoding multifunctional and highly-processed cilium adhesion proteins^16,33–36^ that can also bind glycosaminoglycans^35,37^, extracellular actin^38^, plasminogen^39,40^ and fibronectin^39,41^, all important components of the extracellular matrix of host cells. The ability of *M. hyopneumoniae* to bind to porcine cilia is abrogated by the exogenous addition of the glycosaminoglycan heparin^42^, actin^38^ and by treating cilia with heparinase^43^. Unsurprisingly, *M. hyopneumoniae* adhesins have been explored previously as vaccine targets, though they have proved less effective than the bacterin vaccines currently available^44,45^. This may be due to the growing discovery of *M. hyopneumoniae* surface proteins with adhesion moonlighting capacities^46,47^. Here we describe a novel enzymatic mechanism that potentially enables *M. hyopneumoniae* to inactivate four innate effector peptides that play a central role in controlling ciliary beat frequency, inflammation and other innate immune responses in the lungs. Specifically, we determined that MHJ_0569 and MHJ_0522 are retained on the cell surface of *M. hyopneumoniae* and that purified recombinant versions of these proteases can affect the function of NPY, BK, SP and NKA. rMHJ_0659 was shown to function as a PepP aminopeptidase that removes N-terminal amino acids that lie adjacent to a proline residue in BK, SP, and NPY. rMHJ_0522 (PepF) was shown to function as an oligopeptidase that can cleave BK and the tachykinins NKA and SP that carry a common tachykinin C-terminal motif. These observations provide insight into the mechanisms used by *M. hyopneumoniae* that alter the microenvironment in the upper respiratory tract of the host in a manner that favours colonisation of ciliated epithelium.

Despite lacking translocation signal peptides, MHJ_0659 and MHJ_0522 were identified on the cell surface of *M. hyopneumoniae*. Surface localisation was demonstrated by detecting tryptic peptides released by mild enzymatic cell shaving, digesting biotinylated surface proteins captured by avidin chromatography using LC-MS/MS, and by immunofluorescence microscopy. MHJ_0659 and MHJ_0522 have unique access to host proteins including inflammatory peptides that regulate ciliary function on epithelial cells that line the upper respiratory tract, the preferred niche for this pathogen. Using a MALDI-TOF MS assay, we demonstrate that, collectively, rMHJ_0659 and rMHJ_0522 cleave four peptides that play a central role in modulating mucociliary clearance and inflammation.

BK is a nonapeptide involved in the coagulation cascade, which is now known to be an integral part of innate immunity^20^. Once BK is released from the D4 domain of high molecular weight kininogen via the serine protease kallikrein, it greatly enhances bronchoconstriction, accelerates tracheobronchial and mucociliary clearance, and induces cough^48^. BK binds to mast cells, releasing histamine, leukotriene, and prostaglandins, which lead to increased mucous secretions in the lungs^49^. BK also amplifies nitric oxide production leading to increased ciliary beat frequency^50^ and can initiate a range of other immune responses^51^. rMHJ_0659 cleaved BK at the first two N-terminal amino acids R↓P↓P-G-F-S-P_-_F-R, and rMHJ0522 cleaved BK at R-P-P-G↓F↓S-P-F-R. The cleavage events demonstrated by both proteases mimic those known to inactivate BK^52^.

SP and NKA are neuropeptides belonging to the tachykinin superfamily, characterised by a common C-terminal sequence FXGLM-_NH2_^53^. SP is the most extensively researched tachykinin and is best known for its ability to function as a neurotransmitter. However, it can also induce a repertoire of innate immune effector cells and inflammatory mediators^54,55^, and concentrations of SP are increased within inflamed tissue^56^. Invading bacteria stimulate primary sensory neurons and activate SP NK1 receptors in tissue cells promoting a local influx of inflammatory and immune cells^57^. Additionally, SP has been shown to; (i) increase intracellular calcium which in turn increases ciliary beat frequency^58^; (ii) regulate proinflammatory cytokines^59^; and (iii) stimulate airway submucosal gland secretion^60^. Interestingly, the ability of SP to boost airway submucosal gland secretion is ten-fold greater in pigs than in humans^60^. SP-induced bronchoconstriction and cough have both been reported^61^. However, the ability to induce cough has since been contested^62^. Like SP, NKA induces bronchoconstriction and mucus production in the lungs as well as neurogenic inflammation^63^. Additionally, NKA is a potent attractor of T-cells^57^.

To exert their inflammatory responses, SP and NKA need to bind NK1 and NK2 receptors present in airway smooth muscle, epithelium and on macrophages^64^. rMHJ_0659 cleaved SP at the anticipated N-terminal arginine residue due to the presence of a penultimate proline. While this cleavage does not inactivate SP^65^, it does remove the protective conformation that penultimate proline provides, leaving the peptide vulnerable to potential degradation by other *M. hyopneumoniae* surface exposed aminopeptidases, such as leucine aminopeptidase^47^ and glutamyl aminopeptidase^46^. On the other hand, rMHJ_0522 efficiently cleaved SP at R-P-K-P-Q-Q-F-F↓G-L-M-_NH2._ This cleavage event removes the C-terminal G-L-M-_NH2_ sequence essential for SP to bind tachykinin receptors NK1 and NK2. At pH 7.3 in the presence of Zn^2+^, rMHJ_0522 produced two additional cleavages at R-P-K-P-Q-Q-F-F↓G↓L↓M-_NH2_ thus wholly degrading the G-L-M-_NH2_ sequence. SP fragments are known to be biologically active^66^, and previous reports have demonstrated that F-G-L-M-_NH2_ and G-L-M-_NH2_ can bind NK1 and NK2 receptors, albeit their binding affinity is 1000 times less than that of an intact SP molecule^67^. rMHJ_0522 had a similar activity to SP against NKA producing cleavages at H-K-T-D-S-F↓V↓G↓L-M-_NH2_ again cleaving at the biologically significant C-terminal sequence. Our results indicate that MHJ_0522 can potentially eradicate inflammation mediated by SP and NKA.

NPY is a neuropeptide that has well-recognised roles in the brain; however, NPY has also been identified as a potent mediator of inflammation^68^. Similarly to both BK and SP, NPY increases the release of proinflammatory cytokines and chemokines^69^, but is also a key activator of antigen presenting cell (APC) function^68^. Additionally, allergic airway inflammation in NPY deficient mice is significantly reduced^70^. rMHJ_0659 removed N-terminal tyrosine off NPY. All cofactors tested greatly enhanced rMHJ_04659 activity against NPY at pH 8.8. At a more neutral pH, Ca^2+^ was the most efficient cofactor. The most prominent NPY receptors in the lungs are Y1 receptors, which potentate local inflammatory responses and are also widely expressed on T cells, B cells and APCs^68^. Importantly, studies have demonstrated that removing Y from the N-terminus of NPY diminishes Y1 receptor-binding efficiency and the resultant NPY_2-36_ fragment is a selective Y2 receptor agonist^71^. Y2 receptor agonists have been linked to a reduction in food intake^72^. Our data suggest that cleavage events caused by the action of MHJ_0659 have the potential to minimise Y1 receptor effects and increase Y2 receptor effects.

The data presented here and elsewhere provide a body of evidence to suggest that proteases play an essential role in the pathogenesis of *M. hyopneumoniae* by processing adhesins, lipoproteins and other putative pathogenicity factors^15^, destroying host innate immunity effector peptides and neuropeptides, recycling amino acids for nutrients^42,43^ and by inducing apoptosis^14^. Here we demonstrate novel functions for *M. hyopneumoniae* surface proteases. Our work provides insight into how genome-reduced pathogens colonise and persist in their chosen host.

## Material and Methods

### Materials

Ethylenediaminetetraacetic acid (EDTA), tributylphosphine (TBP), bovine serum albumin (BSA), SP, BK, NKA and NPY were purchased from Sigma (Australia). MS grade trypsin was purchased from Promega (USA). Acrylamide was purchased from Bio-Rad (USA). Immunofluorescence dyes, pre-cast gels, buffers, molecular weight markers and all standard molecular biology reagents were purchased from Life Technologies (Australia) unless otherwise noted.

### Mycoplasma hyopneumoniae culture conditions

*M. hyopneumoniae* strain J was cultured in modified Friss^73^ medium for 48 h at 37 °C and harvested by centrifugation at 12 000 × g for 15 min. Pellets were stored at −80 °C until use.

### Cell surface analyses of *Mycoplasma hyopneumoniae* proteins

All surface exposed *M. hyopneumoniae* proteins were identified using both cell surface biotinylation and trypsin cell surface shaving experiments as previously described^36^. Briefly, for enzymatic cell surface experiments freshly cultured and washed *M. hyopneumoniae* cells were digested with 50 µg/mL trypsin in PBS at 37 °C for 5 min. Any tryptic peptides released were analysed by LC-MS/MS. For surface protein biotinylation, freshly cultured *M. hyopneumoniae* cells were washed and pelleted by centrifugation and then biotinylated with sulfo-NHS-LC biotin (Thermo Scientific) for 30 s. Biotinylated proteins were captured and purified by avidin column affinity chromatography and then separated by 2D SDS-PAGE and examined using LC-MS/MS. A full peptide list of *M. hyopneumoniae* surface proteins^74^ and proteome^75^ have been previously published.

### Antisera generation

Polyclonal antibodies against rMHJ_0659 and rMHJ_0522 were raised in rabbits by the Institute of Medical and Veterinary Science (Australia).

### Western blot

Once SDS-PAGE gel containing a freshly purified recombinant protease sample was electrophoretically transferred, the membrane was washed in PBS with 0.1% Tween [Bio-Rad, USA] for 20 min and then blocked with PBS, 0.1% Tween and 5% skim milk powder for 30 min to prevent non-specific antibody binding. To check specific binding to generated anti-recombinant protease sera, the membrane was then placed in a solution containing primary antibody at a 1:1000 dilution in PBS for 90 min before being washed three times with PBS and probed with peroxidase conjugated anti-rabbit secondary antibody [Sigma Aldrich, USA] diluted to 1:10,000 in blocking solution for 30 min at room temperature. The membrane was again washed briefly three times in PBS before being developed with 3,3′-Diaminobenzidine (DAP) [Sigma Aldrich, USA].

### Immunofluorescence microscopy

Microscopy was performed as previously described^46^ with minimal modifications. A 35 mm WPI Fluorodish was coated with 0.01% poly-l-lysine for 30 min and allowed to air dry for 30 min. An overnight *M. hyopneumoniae* culture was centrifuged (11 000 × *g* for 10 min) and washed twice with sterile PBS. Washed cells were added to the Fluorodish and allowed to attach for 30 min. Excess cells were removed by washing once with PBS followed by fixation in 2% paraformaldehyde at 4 °C overnight. Excess aldehydes were quenched using 100 mM glycine in PBS for 5 min, followed by washing three times with PBS. Non-specific binding sites were blocked with 2% BSA in PBS for 1 h at room temperature, followed by washing three times with PBS. Polyclonal rabbit rMHJ_0522 and rMHJ_0659 antisera were diluted 1:500 with PBS containing 2% BSA, incubated for 1 hr at room temperature and washed three times in PBS. A 1:1000 dilution of goat anti-rabbit antibodies conjugated to CF™ 568 (Sigma-Aldrich) was prepared in PBS containing 2% BSA and incubated for one hr at room temperature and washed three times in PBS. The liquid was removed from the Fluorodish which was allowed to air dry for 15 min. Two drops of VECTASHIELD were added to the Fluorodish followed by imaging on a Nikon Ti inverted epifluorescence microscope, capturing images using a DS-Qi2 microscope camera (Nikon Instruments). These same samples were imaged on a V3 DeltaVision OMX 3D-Structured Illumination Microscopy Imaging System (Applied Precision, GE Healthcare) as previously described^76^. Images were processed using Imaris Scientific 3D/4D image processing software (Bitplane AG).

### Expression and purification of rMHJ_0659 and rMHJ_0522

The *mhj_0659* and *mhj_0522* genes encoding PepP and PepF respectively were ligated into expression vector PS100030, conveying both a hexahistidine tag and ampicillin resistance, by Blue Heron Biotech (USA). All in-frame TGA codons were substituted to TGG. The recombinant construct was transformed into *E. coli* BL21 (Invitrogen) as per the manufacturer instructions and grown overnight in LB supplemented with 100 mg/mL ampicillin. LB supplemented with 100 mg/mL ampicillin was inoculated with overnight transformation culture and grown until mid-log growth phase. Cultures were then induced with IPTG and grown to optimal protein expression levels (4 hrs for rMHJ_0522, 6 hrs for rMHJ_0659). Cells were harvested by centrifugation at 3000 × *g* for 10 min at 4 °C. Pellets were resuspended in Lysis Buffer [50 mM NaH2PO4, 300 mM NaCl, 10 mM imidazole, pH 8) at 2 mL per gram of wet weight and lysozyme (Sigma Aldrich) was added at 1 mg/mL to break down peptidoglycan. Resuspended pellets were incubated on ice for 30 min and then probe sonicated with 6 × 10-sec bursts at 400 W and 10 sec cooling period on ice between bursts. Cell debris was removed by centrifugation at 10,000 × *g* for 30 min at 4 °C and the cleared cell lysate was added to 50% slurry of Profinity immobilized metal affinity chromatography (IMAC) Ni^2+^ charged resin (Bio-Rad) at a volume of 1 mL slurry per 1 L starting culture, and left overnight on a rotary shaker at 4 °C. Both recombinant proteins were then purified under native conditions using imidazole, dialysed against PBS in 10 K MWCO dialysis tubing, and stored at 4 °C. 1D SDS-PAGE was used to separate purified protein samples.

### Proteomics

To analyse purified protein samples separated by 1D SDS-PAGE, peptide preparation by trypsin in-gel digestion, and LC-MS/MS analysis parameters were followed as described previously^46^. A novel assay was used to determine substrate cleavage. From a stock solution (1 mg/ml) of BK, SP, NKA or NPY (Sigma Aldrich) 1 µl was diluted in 8.5 µL 50 mM Tris-HCl buffer (pH 6.3–8.8) or 50 mM sodium acetate (pH 5) and 0.5 µL 100 mM cofactor (Ca^2+^, Co^2+^, Mg^2+^, Mn^2+^, or Zn^2+^). Purified rMHJ_0659 or rMHJ_0522 was added in a 1:20 protease to substrate concentration and incubated for 1 hr at 37 °C. The peptides were then desalted and captured using C18 ZipTips (Millipore). 1 µl of peptide sample was later spotted onto a clean 384-well OptiTOF target plate (AB Sciex) followed by 1 µl of 5 mg/ml α-Cyano-4-hydroxycinnamic acid (CHCA) dissolved in 50% ACN, 0.1% TFA, 10 mM NH_4_H_2_PO_4_ and allowed to dry. Spotted samples were then analysed using a 5800 MALDI-TOF/TOF MS in positive reflector mode for BK, NKA and SP, and linear mode for NPY due to substrate size. Laser intensity was set to 2600 for MS parent ion scans. 400 laser shots were averaged for MS scans. MS parent ion scans were calibrated using a 6-peptide mixture (Sciex). The resulting MS spectral data were then manually inspected to explain the ions present concerning their amino acid sequence and the cleavage events caused by PepP and PepF proteolysis. For inhibition studies, rMHJ_0659 or rMHJ_0522 was pre-incubated with 10 mM metal chelating agent EDTA for 20 min at 37 °C, before repeating substrate cleavage assays using the cofactor that had previously demonstrated highest activity at each pH level tested.
